# Dragon fruit-kiwi fermented beverage: *In vitro* digestion, untargeted metabolome analysis and anti-aging activity in *Caenorhabditis elegans*

**DOI:** 10.3389/fnut.2022.1052818

**Published:** 2023-01-10

**Authors:** Zizhong Tang, Zhiqiao Zhao, Siyi Chen, Wenjie Lin, Qing Wang, Nayu Shen, Yihan Qin, Yirong Xiao, Hong Chen, Hui Chen, Tongliang Bu, Qingfeng Li, Huipeng Yao, Ming Yuan

**Affiliations:** ^1^College of Life Sciences, Sichuan Agricultural University, Ya’an, China; ^2^Sichuan Agricultural University Hospital, Sichuan Agricultural University, Ya’an, China; ^3^College of Food Science, Sichuan Agricultural University, Ya’an, China

**Keywords:** fermented beverage, *in vitro* digestion, untargeted metabolomics, *C. elegans*, antioxidant activity

## Abstract

The research on the development of dragon fruit and kiwi fruit through LAB-yeast compound fermentation is very limited, and there are few related fermentation products on the market. The purpose of this study was to evaluate the stability of the antioxidant capacity of fermented beverages (FB) through *in vitro* simulated digestion, to evaluate the changes in metabolites of juice after fermentation through untargeted metabolomics, and used *Caenorhabditis elegans* as a model to evaluate its anti-aging activity. The results showed that FB not only has good *in vitro* antioxidant activity, but also the total phenol content (TPC), total flavonoid content (TFC), ABTS scavenging ability, and hydroxyl radical scavenging ability of FB were significantly increased during gastric digestion and intestinal digestion. Metabolomics showed that the contents of phenols and flavonoids related to antioxidant increased after fermentation, and fermentation had a significant effect on organic acids and amino acids in FB. Finally, compared with the control group, although the original concentration of FB has a side-toxic effect on nematodes, the mean lifespan of *C. elegans* fed with 1.56% FB increased by 18.01%, SOD activity significantly increased by 96.16% and MDA content significantly decreased by 40.62%. FB has good antioxidant activity *in vitro* and *in vivo*, and the antioxidant activity is stable during the simulated digestion process.

## 1. Introduction

Aging is defined as an irreversible process in which functions of the body decline (1), excessive accumulation of reactive oxygen species is one of the major factors affecting body aging process (2). Therefore, the intake of natural and healthy diets with antioxidant properties has attracted more and more attention, especially the demand for products based on fruits and vegetables is increasing. As we all know, antioxidants such as vitamins, carotenoids and polyphenol compounds contained in fruits and vegetables can enhance the antioxidant defense system and alleviate the accumulation of active oxygen (3). In addition, epidemiological and clinical studies have reported that the consumption of fruits and vegetables containing high antioxidants is beneficial in reducing the risk of cardiovascular disease and cancer (4). However, fruits and vegetables have high moisture content and are very perishable. Probiotic fermentation is a popular preservation method that can improve the nutritional quality and sensory properties of fruit juices (5). Many studies have shown that fruit juices such as pomegranate juice, orange juice and pineapple juice were good substrates for fermentation and production of functional beverages (6–8). During fermentation, the nutrients and compounds in raw food materials will change due to the reaction occur of microorganisms and enzymes, and yeast and lactic acid bacteria (LAB) are common microorganisms in fermented food, the combination of the two will give fermented products good flavor and sensory value (9, 10).

Probiotic functional beverages can not only deliver probiotics to the human intestines, but also meet the consumption such as lactose intolerance, high cholesterol content and milk protein allergy, and the preference of vegetarians (11). In general, this kind of fruit and vegetable juice-based functional beverage has many health-promoting effects, including anti-oxidation, improving the regulation of intestinal flora, and enhancing anti-inflammatory and anti-cancer properties (12). Probiotic functional beverages based on fruit and vegetable juices are popular mainly because of the high contents of ascorbic acid, polyphenols, high amount of sugars that can be metabolized by probiotics (13). In addition, this beverage is considered to be healthy and fresh with suitable taste people of all ages.

Dragon Fruit (*Hylocereus spp*.) is a green, environmentally friendly fruit rich in nutrients such as sugar, protein, organic acids, vitamins, amino acids, and mineral elements (14). It is also a source of trace elements and can be eaten raw or is made into juice, jam, jelly, powder, wine and other products (15). The peel, pulp, and edible seeds of dragon fruit are beneficial to health, such as preventing cancer, anti-inflammatory, reducing cardiovascular disease, and antioxidant (16). Kiwi (*Actinidia chinensis Planch*.) is a kind of fruit, rich in phenolic compounds, triterpenoids, vitamins, dietary fiber and trace elements (17). It can be processed into fruit juice, preserved fruit, yogurt, wine, kiwi slices, fruit and vegetable juice drink vinegar, etc. (17). Ripe kiwi fruit has effects on the spleen, stomach, and kidney meridians, and can improve indigestion, loss of appetite, and vomiting (17). The combined fermentation of a variety of fruits and vegetables can increase the nutrient utilization of the microbial population, thereby affecting its growth and metabolism (18). It can be seen that dragon fruit and kiwi fruit may be good functional food ingredients.

*Caenorhabditis elegans* is an excellent model widely used to study aging. Compared to traditional animal models, *C. elegans* has the advantages of small size, short life span, large progeny production, easy operation (19). In addition, *C. elegans* has been sequenced completely, and 60–80% of human genes have homologs in *C. elegans* (20), making it a major tool for studying the potential health value of food for human chronic diseases. As far as we know, the current research on *C. elegans* is rare to evaluate the effect on antioxidant properties or aging of functional fermented beverages based on fruit and vegetable juices. However, in similar matrices, such as orange juice (21), plant sterol enriched fruit beverages (22) and green coffee extract (2), have proven their beneficial effects on resistance against oxidative stress and delay aging of *C. elegans*. Therefore, the functional fermented beverage based on dragon fruit and kiwi fruit may be a dietary supplement to resist oxidative stress and delay aging.

Although fruit and vegetable fermented beverages have been demonstrated to have many beneficial effects on health, there have been few descriptions of fermented beverages based on dragon fruit and kiwi fruit. Based on this, This study aimed to initially elucidate the mechanism by which fermentation affects changes in antioxidant capacity using non-targeted metabolomic techniques, and further evaluate its stability and effects on growth and anti-stress capacity of *C. elegans*. The research results provide scientific theoretical guidance and reference for the functional evaluation of enzyme food, and have certain novelty.

## 2. Materials and methods

### 2.1. Mixed dragon fruit juice and kiwifruit juice

Fresh dragon fruit and kiwi fruit were purchased from a local supermarket. The fruit is cleaned and cut into small pieces for later use. The mixed juice was prepared by 1 kg of small pieces of fruit (dragon fruit: kiwi, w/w, 1/1) and 3 L of sterile water into the juicer to squeeze and homogenize.

### 2.2. Microorganism and inoculum preparation

Lactic acid bacteria (LAB) and yeasts were donated by Research and Design Institute, Chengdu City, Sichuan Province, China. Considering the risk of mutation and contamination of the strain, LAB and yeast were previously identified by molecular biological methods based on the rigor of the experimental design and the safety of the fermentation products. LAB were identified and named as *Streptococcus thermophilus* strain DR124 and *Leuconostoc mesenteroides* strain DR125, respectively. The yeast was *Saccharomyces cerevisiae* strain Y1. LAB was cultivated in MRS (Man, Rogosa, and Sharpe) medium, and yeasts was cultivated in YPD (Extract Peptone Dextrose) medium. LAB and yeast were inoculated into juice when the concentrations at 5 log CFU/mL and 7 log CFU/mL, respectively.

### 2.3. Preparation of fermented beverage

Add 3 L of fruit juice, 0.84 kg of white sugar to a 5 L glass bottle, and homogenize. The juice was pasteurized (80°C, 5 min), and the juice was immediately cooled with cold water after sterilization. The inoculum of 12% yeast was inoculated into the juice and fermented at 30°C for 15 d. Subsequently, 6% LAB (the *Leuconostoc mesenteroides* and *Streptococcus thermophilus* were mixed in a 1:1 ratio) was inoculated into the juice and fermented at 37°C for 9 d. Then the obtained enzyme solution was centrifuged at 4°C (8900 × *g* for 20 min), and the supernatant was stored at −20°C for later use.

### 2.4. Determination of TPC and TFC

The TPC was determined by the Folin-Ciocalteu method according to Mantzourani et al. (13), with slight modification. 0.5 mL Folin-Ciocalteu reagent and 1 mL 20% Na_2_CO_3_ were added into the 0.5 mL sample. After the mixture is incubated at 75°C for 10 min, the volume is fixed to 5 mL with distilled water. After letting rest at room temperature for 30 min, the absorbance was read at 760 nm by a Microplate Reader (Multiskan Sky, Shanghai Heshang Scientific Instrument Co., Ltd). Standard gallic acid (0.05∼0.035 mg/mL) was used to prepare the standard curve. The TPC was expressed as the weight of gallic acid (mg) per 100 mL sample. Three parallel experiments were conducted.

The TFC was determined by the Aluminum chloride colorimetric method according to Yan et al. (23), with slight modification. 2.5 mL 70% ethanol solution and 0.15 mL 5% NaNO_2_ were added to the 0.5 mL sample, shake and let rest for 6 min, and after that, 0.3 mL of 10% AlCl_3_ solution was added into mixture, stand for 5 min. Finally, 1 mL of 1 mol/mL NaOH was added to the mixture and the volume was fixed to 5 mL with 70% ethanol solution, shaken and let rest for 10 min, the absorbance was read at 510 nm by a Microplate Reader. Standard rutin (0.00∼0.12 mg/mL) was used to prepare the standard curve. The TFC was expressed as the weight of rutin (mg) per 100 mL sample. Three parallel experiments were conducted.

### 2.5. Determination of the antioxidant activity

The antioxidant activity was determined according to the previous method of our research group. The DPPH radical-scavenging activity, ABTS radical-scavenging activity, Hydroxyl radical-scavenging activity was determined according to Zhao et al. (24). Fe^3+^ reducing power was determined according to Tang et al. (25). Three parallel experiments were conducted.

### 2.6. Simulated *in vitro* gastrointestinal (GI) digestion

*In vitro* gastrointestinal (GI) digestion of sample was carried out according to Hou et al. (26), with some modifications.

The simulated gastric fluid (SGF): 0.4 g of NaCl and 0.128 g of pepsin were dissolved in 160 mL distilled water, adjust the pH to 2 ± 0.1 with 0.1 mol/L HCl, the volume is fixed to 200 mL with distilled water. The solution was filtered and sterilized with a 0.22 μm microfiltration membrane. In the process of gastric digestion, the sample was mixed with SGF at a ratio of 1:10 (V:V). The gastric mixture was incubated at 37°C for 2 h at 120 rpm/min, for 0, 0.5, 1, 1.5, 2 h samples are taken and then the TPC, TFC and antioxidant activity of each sample were measured. 0.1 mol/L HCl instead of simulated gastric fluid as the control group. Three parallel experiments were conducted.

The simulated intestinal fluid (SIF): 1.2 g of KH_2_PO_4_ and 2 g of trypsin were dissolved in 160 mL distilled water, adjust the pH to 7.5 ± 0.1 with 0.1 mol/L NaHCO_3_, the volume is fixed to 200 mL with distilled water. The solution was filtered and sterilized with a 0.22 μm microfiltration membrane. In the process of intestinal digestion, the sample was mixed with SIF at a ratio of 1:10 (V:V). The gastric mixture was incubated at 37°C for 2 h at 120 rpm/min, for 0, 0.5, 1, 1.5, 2 h samples are taken and then the TPC, TFC and antioxidant activity of each sample were measured. 0.1 mol/L NaHCO_3_ instead of simulated intestinal fluid as the control group. Three parallel experiments were conducted.

### 2.7. Untargeted metabolomics analysis

#### 2.7.1. Metabolite extraction

The sample was mixed with 80% methanol in a ratio of 1:4 and then vortexed. The sample was placed on ice and incubated for 5 min, then centrifuged (15000 × *g* for 20 min at 4°C), and the supernatant was collected. LC-MS grade water was added to the supernatant to make the final methanol concentration of 53%. Finally, the sample is transferred to a clean Eppendorf tube for centrifugation (15000 × *g* for 20 min at 4°C), and the supernatant is taken for LC-MS analysis.

#### 2.7.2. Instrument parameters

(1) Chromatographic conditions

Hypesil Gold column (Thermo Fisher, 100 × 2.1 mm, 1.9 μm); Column temperature: 40°C; flow rate: 0.2 mL/min. Positive ion mode: Eluent A: 0.1% formic acid; Eluent B: Methanol; Negative ion mode: Eluent A: 5 mM ammonium acetate (pH 9.0); Eluent B: Methanol. The same chromatographic gradient elution procedure in both ion modes. Gradient which consisted of 2% eluent B, 1.5 min; 2-100% eluent B, 12.0 min; 100% eluent B, 14.0 min; 100-2% eluent B, 14.1 min; 2% eluent B, 17 min.

(2) Mass spectrometry conditions

The mass spectrometer was operated in positive/negative polarity mode with the following settings: Sheath gas flow rate: 40arb. Aux Gasflow rate: 10 arb. Spray Voltage: 3.2 Kv. Capillary Temp: 320°C.

#### 2.7.3. Database search

The Compound Discoverer 3.1 (CD3.1, Thermo Fisher) was used to process the raw data via retention time, mass-to-charge ratio and other parameters, and then perform peak alignment, peak extraction and peak area quantification for metabolites. Match the data obtained from the above analysis with mzCloud, mzVault and Masslist databases to obtain metabolite identification and relative quantitative results.

### 2.8. Antioxidant activities of FB *in vivo*

#### 2.8.1. Culture and treatment of *C. elegans*

Wild-type *C. elegans* N2 and *Escherichia coli* OP50 was given by the Department of Botany, College of Life Sciences, Sichuan Agricultural University. The nematodes were cultured on NGM medium at 20°C with *E. coli* OP50 as food. Synchronized nematodes were obtained through the sodium hypochlorite method according to the previous method (27).

#### 2.8.2. Assessment of acute toxicology

Toxicity tests according to Moliner et al. (28), with slight modification. Add K solution (32 mM KCl, 51 mM NaCl) to FB to make the final concentration of FB 100%, 50%, 25%, 12.5%, 6.25%, 3.13%, 1.56% (V:V). Nematodes were subjected to assess of acute toxicology in a microtiter plate. 100 μL of sample solution of different concentrations per well to treat nematodes, 30 individuals per treatment. After 24 h of incubation at 20°C, count and record the number of dead nematodes. The result is expressed in survival rate%. Survival rate% = (Number of live nematodes/Total number of nematodes)*100. K solution as a positive control.

#### 2.8.3. Lifespan assays

After the lysis of sodium hypochlorite, the eggs were hatched in fresh NGM medium coated with different concentrations of FB (indicates adding 25%, 6.25%, 1.56% of FB) or without FB (control, indicates *E. coli* adding 0% of FB) and incubated to the L4 stage under these conditions. The nematodes were transferred to fresh NGM every day, and the number of live nematodes were counted. The survival rate is calculated as: number of live nematodes/(number of live nematodes + number of dead nematodes)*100. Each group of 90 nematodes was performed in parallel, and the experiment was repeated three times.

#### 2.8.4. Fertility assay and body bending frequency

The synchronization method of nematodes has been described in the lifespan assays. The nematodes of L4 stage were transferred to fresh NGM every day, and the number of eggs were counted. Each group of 10 nematodes was performed in parallel, and the experiment was repeated three times.

The body bending frequency was observed according to Wang et al. (29).

#### 2.8.5. Oxidative stress and heat stress

The synchronization method of nematodes has been described in the lifespan assays. The FB-treated nematodes were placed on an NGM plate containing 2 mM H_2_O_2_ for 5 h for an acute oxidative stress experiment, and incubated at 35°C for 5 h for an acute heat stress experiment. Record the number of survivors after the treatment. In the absence of any reaction, determine the death of the worm by touching the head and tail of the worm. Each group of 90 nematodes was performed, and the experiment was repeated three times.

#### 2.8.6. Determination of antioxidant enzyme activity and MDA content *in vivo*

After pretreatment of worm N2 with 6.25% FB for 5 days, it was wash from NGM with M9 buffer. Next, the nematodes were crushed by ultrasound to obtain a homogenate, and finally the homogenate was centrifuged (8900 × *g* for 20 min at 4°C) (Thermo Scientific Sorvall ST 16, Shanghai Fuze Trading Co., Ltd), and the supernatant was taken to measure the protein content and MDA content, SOD activity according to the corresponding kit (Jiancheng, Nanjing, China).

### 2.9. Statistical analysis

Statistical analysis was carried out using the SPSS 22 software (SAS Institute Inc., USA). Analysis of variance (ANOVA) was used to evaluate the significant difference among various treatments. Use the KEGG database^1^ to study the functions of metabolites and metabolic pathways; Use metaX to perform principal component analysis (PCA) and partial least squares discriminant analysis (PLS-DA) to obtain the variable importance in the project (VIP) value of each metabolite; Use univariate analysis (*t*-test) to calculate statistical significance (P-value); Set the standard as VIP > 1, *P* < 0.05 and (fold change) FC ≥ 2 or FC ≤ 0.5 to maximize the identification of differential metabolites; Use the R package ggplot2 to draw volcano graphs and bubble graphs; Use R package Pheatmap to draw clustering heat map.

## 3. Results and discussion

### 3.1. Evaluation antioxidant activity of FB

As a functional product, the evaluation of the antioxidant activity of FB is very important for product promotion. However, there is currently no standard method for evaluating the antioxidant properties of foods and beverages. It is recommended to use two or more methods in combination to provide information on the antioxidant capacity of beverages. In this study, TPC, TFC, three radical scavenging ability (DPPH, ABTS, Hydroxyl radicals) and Fe^3+^ reducing power were selected to evaluate the antioxidant activity of FB. The results were shown in Table 1. The DPPH and Hydroxyl radicals scavenging activity and reached as high as about 90% in FB, suggesting that FB has a good potential for free radical inhibitory activity, reflecting good antioxidant activity potential.

**TABLE 1:** FB antioxidant activity.

	TPC (mg/mL)	TFC (mg/mL)	DPPH scavenging rate (%)	ABTS scavenging rate (%)	Hydroxyl radicals scavenging rate (%)	Fe^3+^reducing power
Fruit juice	0.052±0.001	0.041±0.002	46.46±1.327	38.21±1.16	46.27±2.945	0.882±0.017
FB	0.062±0.002	0.161±0.008	91.55±0.442	62.02±1.67	93.60±1.535	0.702±0.006

### 3.2. Simulated *in vitro* gastrointestinal (GI) digestion

Although FB has good antioxidant capacity, whether it has high antioxidant activity after being digested and absorbed by the body was a question that people are eagerly concerned about. FB was exposed to SGF and SIF.

#### 3.2.1. The effect of *in vitro* simulated gastrointestinal digestion on TPC and TFC of FB

**FIGURE 1:**
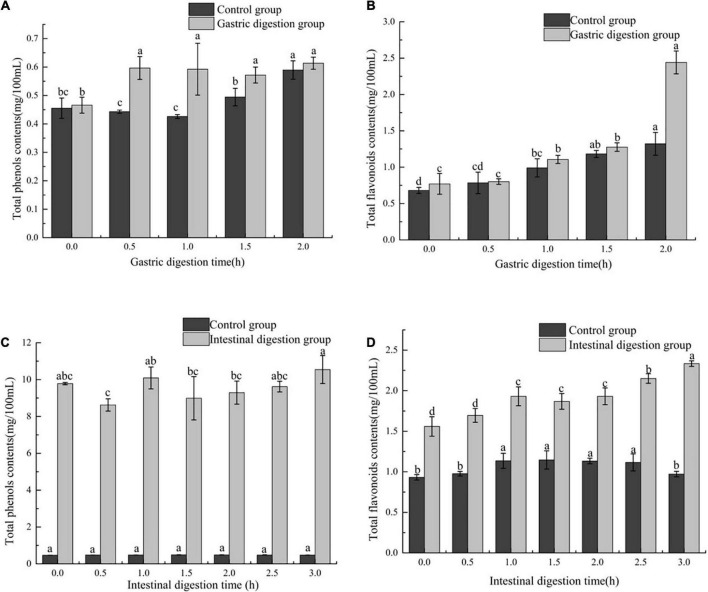
The effect of *in vitro* gastrointestinal (GI) digestion on TPC and TFC. **(A)** The effect of *in vitro* gastric digestion on TPC, **(B)** the effect of *in vitro* gastric digestion on TFC, **(C)** the effect of *in vitro* intestinal digestion on TPC, and **(D)** the effect of *in vitro* intestinal digestion on TFC. Different lowercase letters indicate significant differences (*p* < 0.05), and the same characters are not significantly different.

The phenolic compounds and flavonoids compounds are a natural antioxidant found in plant foods. In order to evaluate the effect of *in vitro* digestion on phenols and flavonoids in FB, TFC and TPC during the digestion of FB were determined. During the gastric digestion stage, the contents of total phenols and total flavonoids in both the digestion group and the control group of FB showed an upward trend with time (Figures 1A, B). The TPC in the digestion group increased by 29.79%, and the control group increased by 28.26% (Figure 1A). TFC in the digestion group increased from 0.77 ± 0.142 mg/100 mL to 2.44 ± 0.157 mg/100 mL, and the control group increased from 0.68 ± 1.322 mg/100 mL to 1.32 ± 0.157 mg/100 m L (Figure 1B). The presence of an acidic environment affects some of the bound phenols in FB, such as polyphenol proteins bound by hydrogen bonds or amide bonds or polyphenol carbohydrate complexes bound by hydrophobic bonds, and release phenolic substances through bond breakage or hydrolysis come out (30). The presence of digestive enzymes reduces the ester bond between part of the phenolic acid and the cell wall to release more free acid (31). This observation is consistent with the reported by Chen et al. (32), who report TPC of some of fruits increases after gastric digestion, which is about 47.97 times the initial value. The TFC of the gastric digestion group and the control group increased by 68.41% and 48.49%, respectively (*P* > 0.05), and the TFC of the digestion group was consistently higher than that of the control group (Figure 1B), indicating that the flavonoids in FB are stable in the presence of pH and pepsin.

In the intestinal digestion stage, the alkaline environment had no significant effect on the TPC of FB, while trypsin affected the total phenolic content. It can be seen from Figure 1C that the TPC of FB has no significant change between 1 and 2.5 h, and reaches the maximum value at 3 h, which is 10.55 ± 0.759 mg/100 mL, an increase of 7.87% compared with the initial. The increase in trypsin hydrolysis activity in the intestinal digestion group promotes the release of phenolic compounds bound to the matrix (33). In this case, the newly produced phenolic compounds are more than the phenolic compounds degraded due to alkaline environment, thus increasing the content of TPC in the intestinal digestive. Interestingly, the TFC of FB in the intestinal digestion group was also higher than the TFC of FB in the gastric digestion group. With the prolongation of digestion time, the TFC in the intestinal digestion group and control group of FB increased, from 1.56 ± 0.119 to 2.33 ± 0.034 mg/100 mL in the digestion group, and from 0.93 ± 0.034 mg/100 mL in the control group. 0.034 mg/100 mL increased to 0.97 ± 0.034 mg/100 mL (Figure 1D). The TFC of the intestinal digestion group was higher than that of the gastric digestion group. However, Yan Y et al. reported the TFC of the fermented blueberry pomace was decreased after intestinal digestion (23). This is because the flavonoids undergo fission during the digestion process and the ring is degraded to form phenolic acids (34).

#### 3.2.2. The effect of *in vitro* simulated gastrointestinal (GI) digestion on antioxidant activity of FB

**FIGURE 2:**
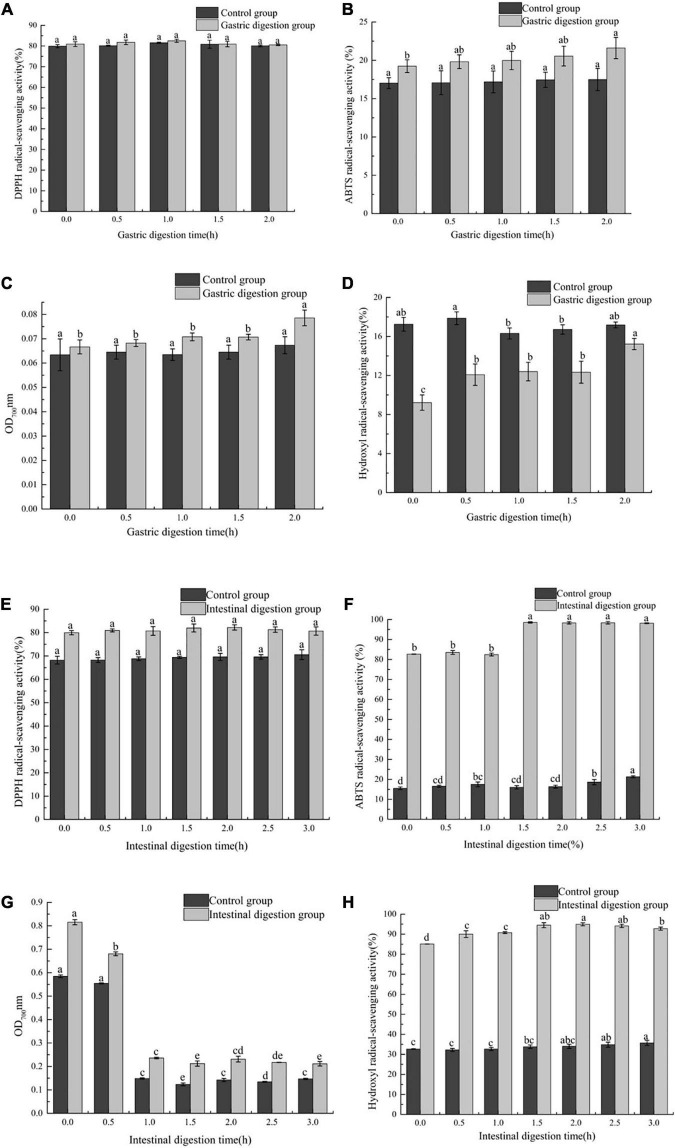
The effect of *in vitro* gastrointestinal (GI) digestion on antioxidant activity. **(A)** The effect of *in vitro* gastric digestion on DPPH radical-scavenging activity, **(B)** the effect of *in vitro* gastric digestion on ABTS radical-scavenging activity, **(C)** the effect of *in vitro* gastric digestion on Fe^3+^ reducing power, **(D)** the effect of *in vitro* gastric digestion on Hydroxyl radical-scavenging activity, **(E)** the effect of *in vitro* intestinal digestion on DPPH radical-scavenging activity, **(F)** the effect of *in vitro* intestinal digestion on ABTS radical-scavenging activity, **(G)** the effect of *in vitro* intestinal digestion on Fe^3+^ reducing power, and **(H)** the effect of *in vitro* intestinal digestion on Hydroxyl radical-scavenging activity.

Four different antioxidant experiments (DPPH free radical scavenging ability, hydroxyl free radical scavenging ability, ABTS free radical scavenging ability, Fe^3+^ reducing power) were used to study the effect of *in vitro* simulated gastrointestinal (GI) digestion on antioxidant activity of FB. In the gastric digestion stage, in the DPPH, ABTS and Fe^3+^ reducing power assays, the antioxidant capacity of the gastric digestion group was stronger than that of the control group, and at this stage, the control group had no significant difference (*P* > 0.05). The scavenging ability of FB on DPPH has been maintained at about 80% (*P* > 0.05) (Figure 2A). Compared with the ability to scavenge DPPH, the ability of FB to scavenge ABTS was lower, about 20% (Figure 2B). At 2 h, the ability of FB to scavenge ABTS reached the maximum, which was 21.61 ± 1.29%. The reducing power of FB on Fe^3+^increased from 0.067 ± 0.003 to 0.079 ± 0.003 (Figure 2C). In the hydroxyl radical experiments, the antioxidant capacity of the control group was stronger than that of the digestive group. At this stage, the hydroxyl radical scavenging ability of the digestion group increased by 39.42% (*P* < 0.05) compared with the initial period, and control group reached the maximum at 0.5 h, which was 17.89 ± 0.65% (Figure 2D). However, in the intestinal digestion stage, in the four antioxidant experiments, the antioxidant capacity of the intestinal digestion group was stronger than that of the control group. There was no significant difference in the scavenging activity of FB on DPPH between the control group and the intestinal digestion group (*P* > 0.05) (Figure 2E). In the intestinal digestion group, the scavenging ability of FB on hydroxyl free radicals reached more than 85% (Figure 2H), and the scavenging ability of ABTS free radicals reached above 80% (Figure 2F), the Fe^3+^ reducing power was reduced from 0.815 ± 0.011 to 0.211 ± 0.009 (Figure 2G).

In this study, FB showed different antioxidant effects at different digestion stages. This may be because in the *in vitro* digestion process, the antioxidant compounds undergo chemical changes, thereby changing their chemical properties and functions, resulting in different antioxidant activity results (35). Secondly, different antioxidant compounds have different mechanisms of action, and each compound has its specific target in the reaction matrix (36). Therefore, different chemical reactions may lead to different degrees of antioxidant capacity in various chemical tests. In the intestinal digestion stage, the scavenging ability of FB on hydroxyl free radicals and ABTS free radicals and Fe^3+^ reducing power were stronger than its effect in the gastric digestion stage, and the trend of the three changes was the same as the trend of TPC. These results were in accordance with those reported by Attri et al. (37), who noted that after simulating the digestion of the gastrointestinal tract, the release of polyphenols in the juice was mainly achieved in the intestinal phase. This phenomenon may be related to the release of protein-bound phenolic substances and the transition from an acidic environment to an alkaline environment, because the hydroxyl groups in the aromatic ring are deprotonated, which finally leads to the enhancement of the antioxidant capacity of phenolic compounds in the gastrointestinal simulation (38). In addition, Bermúdez-Soto et al. (39) reported that the higher antioxidant activity in the intestinal environment may be due to the biotransformation of polyphenol compounds into other phenolic compounds and antioxidant substances under mild alkaline conditions. The structural complexity and degree of polymerization of polyphenols affect their absorption sites. Low molecular weight polyphenols with monomer and dimer structures are easily absorbed by the gastrointestinal tract, while other polyphenols are easily absorbed by the intestine or other parts of the digestive tract (40). In different digestion processes (gastric digestion and intestinal digestion), FB has stable antioxidant capacity, which indicates that FB can be used as a good source of antioxidants, although its antioxidant activity was low at a certain stage.

### 3.3. Data quality evaluation of untargeted metabolomics analysis

In order to understand the difference of metabolites before and after FB fermentation and to preliminarily clarify the mechanism of the change of antioxidant capacity of FB. In this study, non-targeted metabolomics techniques were used to analyze pre-fermentation (DKc) and post-fermentation (DKs) samples of FB.

#### 3.3.1. Sample quality (QC) control

The pearson correlation coefficient between QC samples is calculated based on the peak area value of metabolites. The results showed that QC samples were highly correlated, and the data quality was high (Supplementary Figure 1).

#### 3.3.2. Principal component analysis (PCA) and partial least squares discrimination analysis (PLS-DA) results in the DKc and DKs

**FIGURE 3:**
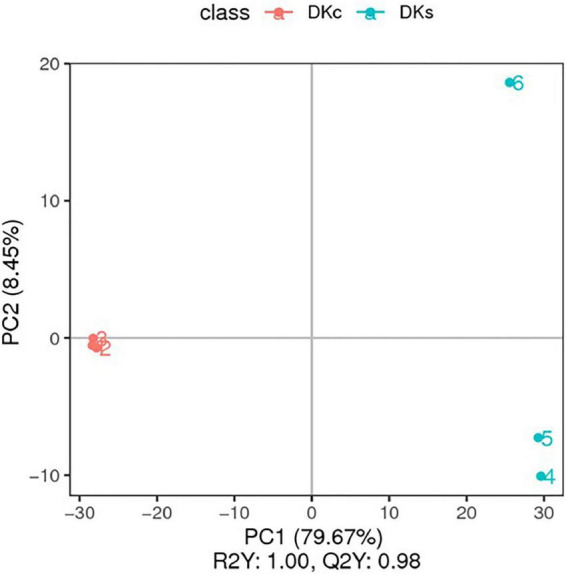
The PLS-DA. The abscissa is the score of the sample on the first principal component; the ordinate is the score of the sample on the second principal component; R2Y represents the interpretation rate of the model, and Q2Y is used to evaluate the predictive ability of the PLS-DA model, and when R2Y is greater than Q2Y Indicates that the model is well established.

The PCA scores plot showed that the results of the three treatment groups have a good separation effect between DKc and DKs in the two modes (Supplementary Figure 2). The PLS-DA score map also gets a clear separation effect (Figure 3). DKc was distributed on the negative axis of the coordinate axis, and DKs was distributed on the positive axis of the coordinate axis, indicating that there was a significant difference between DKc and DKs in the accumulation of metabolites. In addition, the ranking verification results show that the PLS-DA model was not over-fitting (Supplementary Figure 3). The criterion is: R^2^ value was greater than Q^2^ value, and the Q^2^ regression line intersects with the Y axis and is 0 or below (41). Therefore, the model established in this research is accurate and reliable, and can be used for subsequent data analysis.

#### 3.3.3. Volcano plots of differential metabolites

**FIGURE 4:**
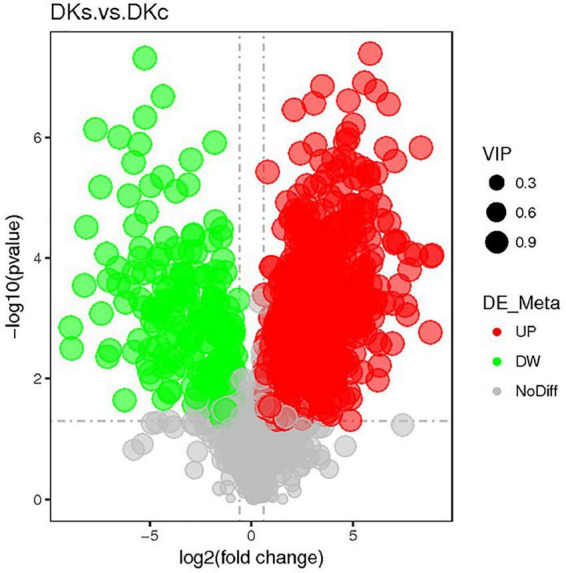
The volcano plot of the different metabolites in different samples. The abscissa represents the fold change of metabolites in different groups (log2 Fold Change), the ordinate represents the significant level of difference (-log10*p*-value), and each point in the volcano map represents a metabolite.

The volcano maps showed the overall distribution of endogenous differential metabolites. In the volcano map (Figure 4), the gray dots indicated metabolites that are not significantly different in the DKc group and the DKs group, and the red dots and green dots indicate the metabolites that are significantly up-regulated and significantly down-regulated in the DKs group, respectively. There were 825 differentially abundant metabolites in DKc and DKs, of which 640 were significantly up-regulated and 185 were significantly down-regulated. The active substances with significantly increased content of differential metabolites included: Riboflavin (FC 6.56), m-Cresol (FC 27.99), Hesperetin (FC 4.62), Sinapyl Alcohol (FC 32.09), N-Feruloyltyramine (FC 79.72), Catechin (FC 12.73), Isorhamnetin (FC 17.85), Nobiletin (FC 37.86), Isoberberine (FC 214.07), Dihydrokaempferol (FC 6.81) and Epigallocatechin (FC 14.49). The increase in the content of these phenols or flavonoids played a great role in improving the antioxidant capacity of FB.

#### 3.3.4. Metabolite heatmaps in the DKc group and DKs group

**FIGURE 5:**
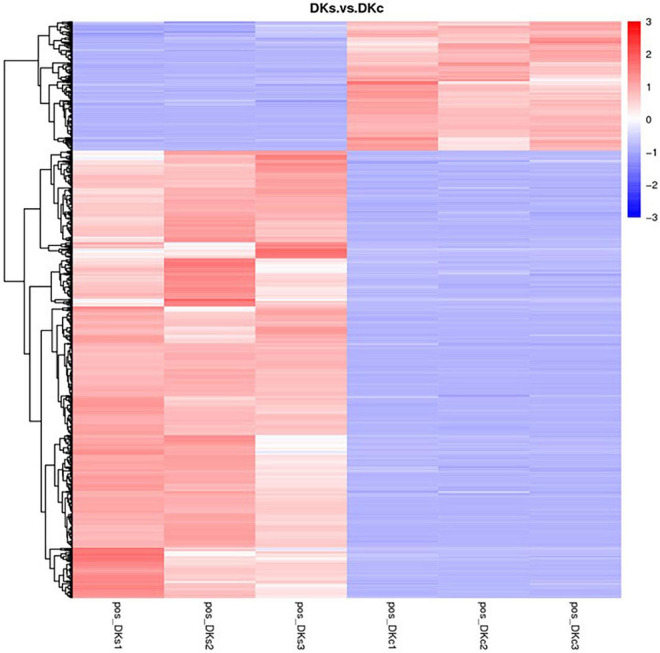
The metabolites heatmap between the DKc group and DKs group. The threshold was set as VIP > 1.0, FC > 1.5 or FC < 0.667, and *P*-value < 0.05. Up expression and down expression are presented in red and blue, respectively.

In this study, metabolites heatmaps were obtained to analyze the metabolic patterns of DKc group and DKs group. The colors in the cluster heat map represent the relative content of metabolites in this group of samples, where the color changes from blue to white and then to red, indicating that the content of metabolites in the samples is up-regulated. It can be seen from Figure 5 that the number of metabolites with increased content was higher than that with reduced content after fermentation.

#### 3.3.5. The KEGG pathway

**FIGURE 6:**
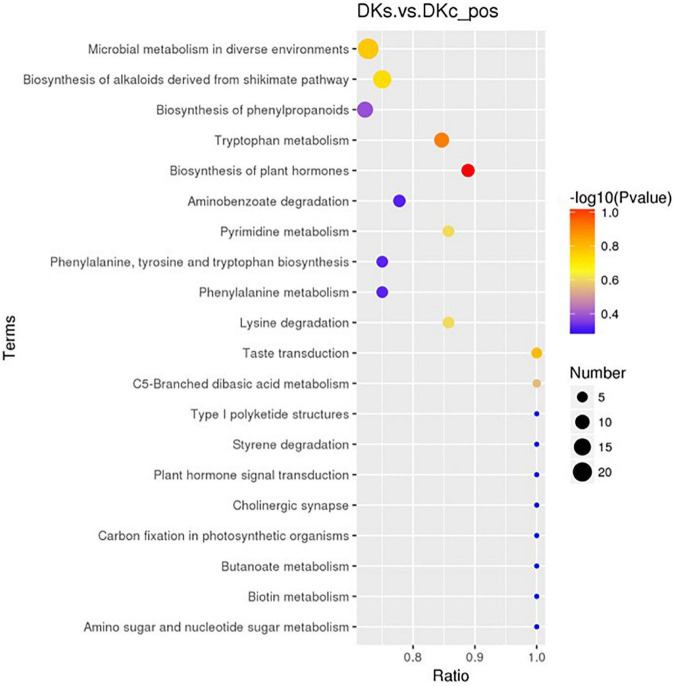
The Kegg pathway that the significant different metabolites take part in between the DKc group and DKs group. The abscissa is x/y (the number of differential metabolites in the corresponding metabolic pathway/the total number of metabolites identified in the pathway). The larger the value, the higher the enrichment of differential metabolites in the pathway. The color of the dot represents the p-value of the hypergeometric test. The smaller the value, the greater the reliability of the test and the more statistically significant. The size of the dot represents the number of different metabolites in the corresponding pathway. The larger the dot, the more differential metabolites in the pathway.

A total of 83 differential metabolic pathways were obtained by pathway enrichment analysis. The top 20 metabolic pathways were shown in Figure 6. The top 5 metabolic pathways were: (i). Biosynthesis of plant hormones, 8 metabolites such as shikimic acid, aspartic acid and L-phenylalanine were found in this pathway. (ii). Tryptophan metabolism, 11 metabolites such as Serotonin, Indole, Anthranilic acid were found in this pathway. (iii). Taste transduction, 5 metabolites such as Serotonin, Gamma-Aminobutyric acid, D-Phenylalanine were found in this pathway. (iv). Microbial metabolism in diverse environments, 24 metabolites such as Vitamin C, Syringic acid, Protocatechuic acid, Gamma-Aminobutyric acid, Gluconolactone, L-lysine were found in this pathway. (v). Biosynthesis of alkaloids derived from shikimate pathway, 18 metabolites such as Dictamnine, Shikimic Acid, Catharanthine were found in this pathway. Among them, L-phenylalanine, an aromatic amino acid with pharmacological activity and one of the essential amino acids for the human body, is mainly used in sweeteners, flavoring agents and health products in the food industry (42, 43). GABA, a non-protein amino acid, is one of the main inhibitory neurotransmitters in the central nervous system, it has physiological functions such as neurotransmission, inducing hypotension, diuresis, and sedation (44). In addition to antioxidant and antibacterial effects, organic acids also have other pharmacological activities. Shikimic acid has 79 biological activities such as anti-inflammatory, analgesic, and anti-thrombotic activities (45). Ascorbic acid (Vitamin C) is good for treating various diseases such as bronchitis, gastric ulcer, cataract, dysentery, Alzheimer’s disease, depression, as well as for repairing Bones and teeth are also effective (46). Syringic acid, a kind of phenolic acid, can significantly improve the liver oxidative and inflammatory damage induced by ethanol (47). Vasicine and protocatechuic acid can be used as the main active ingredients of analgesic, anti-inflammatory, antibacterial, anti-tumor, anti-oxidant and other drugs (48). In addition, prototheranic acid can also relieve obesity-causing disorders (48). Serotonin is a neurotransmitter in the central nervous system (CNS), involved in many central nervous system diseases, such as depression, anxiety, schizophrenia, obsessive-compulsive disorder, addiction, Parkinson’s disease, and peripheral organ diseases, such as gastrointestinal Disease, arrhythmia, high blood pressure (49). Gluconolactone combines with water to inhibit the loss of epidermal moisture and achieves the effect of retaining water and beautifying the skin (50). In addition, it can also reduce the number of inflamed and total lesions to improve acne in patients (51).

Through non-targeted metabolomic analysis, we initially found that fermentation increased the content of antioxidants (phenols and flavonoids) compared with unfermented juice, and fermentation had a greater impact on organic acids and amino acids in FB. In addition, we also found that the active molecules contained in FB can enrich the nutritional value of FB, making it have the potential to develop into functional FB. It has good application market and promotion prospects in beauty, food, medical and other fields. However, the mechanisms of these aspects are still unclear and worthy of continued research.

### 3.4. *C. elegans* assays

#### 3.4.1. Evaluation of FB acute toxicity in *C. elegans*

As a food, FB is very important for its safety evaluation. The acute toxicity of the FB was evaluated by studying their effect on the survival rate of *C. elegans*, which is a widely used and accepted animal model in the field of toxicology due to the similarity between nematodes and mammals (52). The treatment with FB at a concentration of 50∼1.56% for 24 h did not affect the viability of the nematodes when compared with the control (Supplementary Figure 4) (*P* > 0.05), and the survival rate was more than 94% (V/V). At the maximum concentration (100%), the survival rate of nematodes was significantly reduced by 43.95% compared with the control. This result showed that the low concentration of FB has no toxic and side effects on nematodes, that is, it is safe. For this reason, we performed the following experiments using 25%, 6.25%, and 1.5%. Although the original concentration of FB has a bytoxic effect on nematodes, this result is not inconsistent with the good antioxidant capacity of the original concentration of FB, because the components that play a role in the two experimental phenomena are different.

#### 3.4.2. Extended lifespan of *C. elegans* by FB under normal culture condition

**FIGURE 7:**
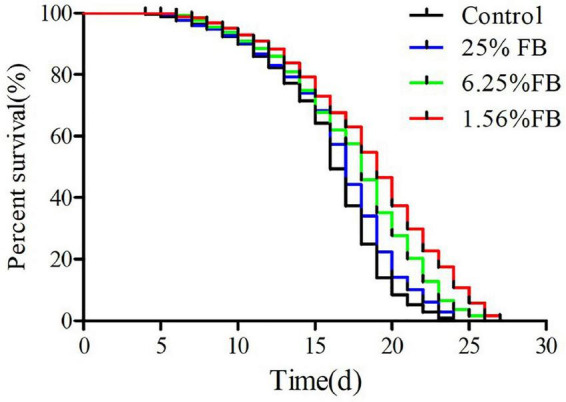
The effects of FB on the lifespan of *C. elegans*.

The lifespan curve of nematodes was shown in the Figure 7 and the lifespan statistics were shown in Table 2. After administration, compared with the control, the lifespan curve of nematodes was shifted to the right, and the mean lifespan was extended by 4.29%, 10.60%, and 18.01%. The results showed that FB extended lifespan of *C. elegans*. Previous studies have shown that antioxidants such as vitamins A, C, E, resveratrol, etc. play an important role in extending life and extending healthy life (53). In this study, the metabolome data showed that the relative content of most antioxidants such as Sequoyitol, Isorhamnetin, Resveratrol, Procyanidin A3, Vitamin B1, Isosinensetin, increased after fermentation. Therefore, the extended lifespan effect of FE may be related to the increase of these antioxidants. In this study, 1.56% FB was selected for the subsequent *in vivo* testing of worms because this concentration has the best extended lifespan effect.

**TABLE 2:** The effect of FB on the lifespan of *C. elegans.*

Concentration (%, v/v)	Number of worms	Mean lifespan±SEM(d)	*P*-value^a^	Effect (%)^b^
FB (0%)	249	15.38±0.35	–	–
FB (25%)	246	16.04±0.20	0.0124	4.29%
FB (6.25%)	242	17.01±0.21	<0.0001	10.60%
FB (1.56%)	241	18.15±0.28	<0.0001	18.01%

^a^*P*-value was calculated using the Kaplan-Meier survival analysis, Log-rank test against by comparing each concentration of the FB-treated group with control (0 mg/L of FB).

^b^% effect was calculated by increase mean lifespan compared to control (0 mg/L of FB).

#### 3.4.3. FB showed no effect on the fertility and body bending of *C. elegans*

In order to evaluate the effect of FB on the fertility of nematodes, the number of progeny of nematodes was investigated. The result was shown in Supplementary Figure 5A. The number of progeny reached the maximum on the day 3, and then gradually decreased until it was completely over on the day 7. After administrating with 1.56% FB, the number of progeny of the nematodes increased by 3.58% compared with the control group, but there was no significant difference (*P* > 0.05). This result indicates that FB nematode fertility has no effect.

The motor behavior of nematodes can reflect the basic functions of the nervous system, and the abnormal motor state can reflect the defects of nerve or muscle function (29). As shown in Supplementary Figure 5B, in terms of worm bending frequency, there was no significant difference between the control group and the 1.56% FB treatment group, indicating that FB has no effect on worm exercise capacity.

#### 3.4.4. FB increased antioxidant enzyme activity and reduced MDA content in *C. elegans* under normal culture condition

**FIGURE 8:**
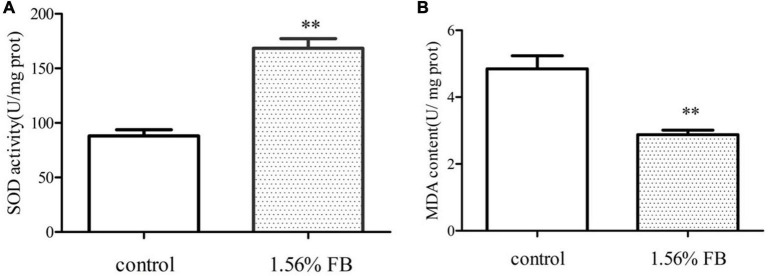
Effect of FB on the activities of antioxidant enzymes. **(A)** SOD activity and **(B)** MDA content. Differences compared to the control group were considered significant at **p < 0.01.

Antioxidant enzyme system is one of the most important defense lines to resist oxidative stress and eliminate active oxygen in the cells. SOD is antioxidant enzymes. MDA is one of the important products of membrane lipid peroxidation and a common indicator of aging physiology and resistance physiology. Compared to the control group, the SOD activity of the FB treatment group was significantly increased by 96.16% (*P* < 0.01) (Figure 8A) and the MDA content was reduced by 40.62% (Figure 8B).

#### 3.4.5. FB increased oxidative stress tolerance and heat stress tolerance in *C. elegans*

Typically, there was a strong positive correlation between life extension and the survival rate of nematodes under stress (54). Free radicals are excessively produced under heat stress, and its antioxidant effect can be effectively evaluated by detecting the protective effect of samples on nematodes under heat stress (29). Compared with the control, the survival rate of nematodes in the 1.56%FB treatment group was increased by 19.24% and 16.94% under oxidative stress and heat stress conditions, respectively (Supplementary Figures 6A, B). Therefore, FB can improve the resistance of nematodes under oxidative stress and heat stress, and has a good antioxidant capacity *in vivo*.

Health and longevity are the eternal pursuit of human subjects. Although aging is irreversible process, dietary intervention can effectively fight aging and improve health. Reports revealed that raspberry extract ameliorates oxidative stress in *C. elegan*s via the SKN-1/Nrf2 pathway (1), and plant sterol and galactooligosaccharides enriched beverages enhance oxidative stress and longevity in *C. elegan*s (22). Dragon fruit and kiwi fruit are rich in a variety of functional ingredients, but there are few studies on their longevity and anti-stress in *C. elegans*, especially on their fermented products. In this study, we found that low concentrations of FB were safe for nematodes. In addition, the activities of SOD in nematodes treated with FB were increased and the content of MDA was decreased, indicating that FB may delay the aging by increasing the antioxidant enzymes in the nematodes and reducing the content of MDA.

## 4. Conclusion

Functional fruit fermented beverages are popular at all ages due to their health effects. However, research on the development of dragon fruit and kiwi fruit by compound fermentation was very limited, and there are few related fermentation products on the market. In this study, a FB was obtained by two-step fermentation of yeast and lactic acid bacteria. It was found that the FB had good antioxidant capacity *in vitro*, and the antioxidant capacity was relatively stable during simulated digestion *in vitro*. In addition, with the aid of metabolomic dates we also found that phenolic and flavonoid contents were increased after fermentation. Finally, within the safe concentration range, this fermented beverage has no effect on the number of progenies by the nematodes, the frequency of body bending, and can extend the life of the nematodes and have a better survival rate under stress.

## Data availability statement

The original contributions presented in this study are included in the article/Supplementary material, further inquiries can be directed to the corresponding authors.

## Author contributions

ZT and ZZ designed and performed the experiments. SC, WL, QW, NS, and YQ analyzed the data and drew diagrams and tables. YX, HoC, and HuC drafted the work. HuC supervised and administrated the work. TB, QL, HY, and MY revised it critically for important content. All authors contributed to the article and approved the submitted version.

## Footnotes

1
https://www.genome.jp/kegg/pathway.html

## Supplementary material

Supplementary Figure 1 QC sample correlation analysis.

Supplementary Figure 2 PCA analysis.

Supplementary Figure 3 The PLS-DA ranking test.

Supplementary Figure 4 Effects of FB on the survival rate of wild-type *C. elegans*. Differences compared to the control group were considered significant at p < 0.0001 (****) and ns (not significance differences).

Supplementary Figure 5 The effects of FB on the number of progeny **(A)** and body bending **(B)** of *C. elegans.* No significant (ns) difference compared with the control group.

Supplementary Figure 6 Effect of Oxidative stress **(A)** and heat stress (**B**) on survival rate of *C. elegans.* Differences compared to the control group were considered significant at p < 0.05(*).
