# Supplementary figures and images for: Dragon fruit-kiwi fermented beverage: In vitro digestion, untargeted metabolome analysis and anti-aging activity in Caenorhabditis elegans
Source: Front Nutr. 2023 Jan 10;9:1052818. doi: 10.3389/fnut.2022.1052818 (PMC9872153; doi:10.3389/fnut.2022.1052818)

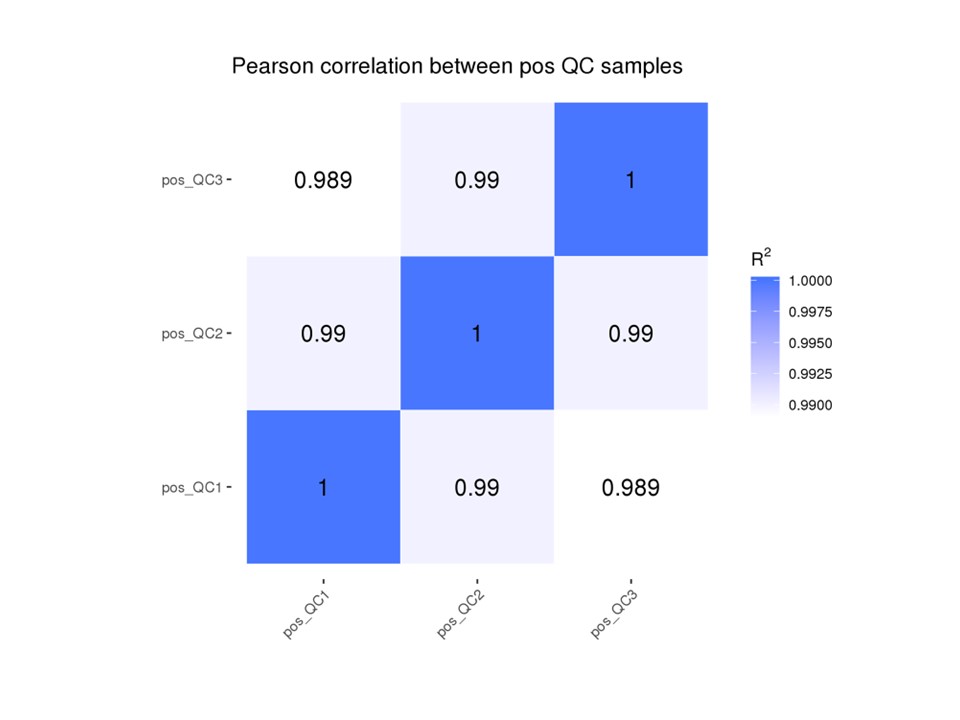

Supplement: Supplementary Figure 1 — QC sample correlation analysis. [file Image_1.JPEG]

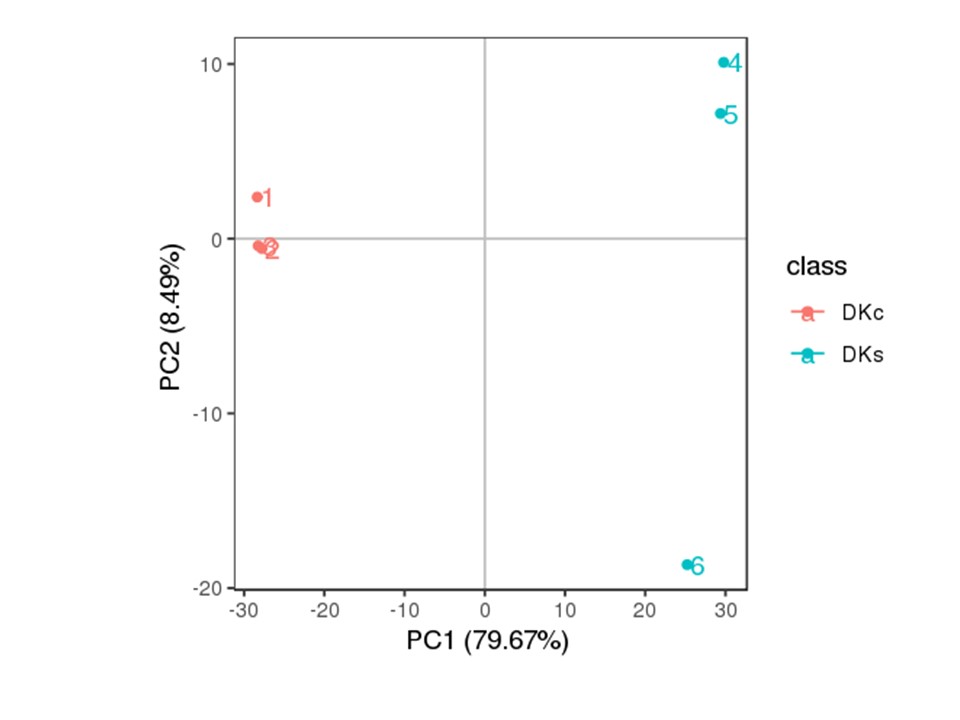

Supplement: Supplementary Figure 2 — PCA analysis. [file Image_2.JPEG]

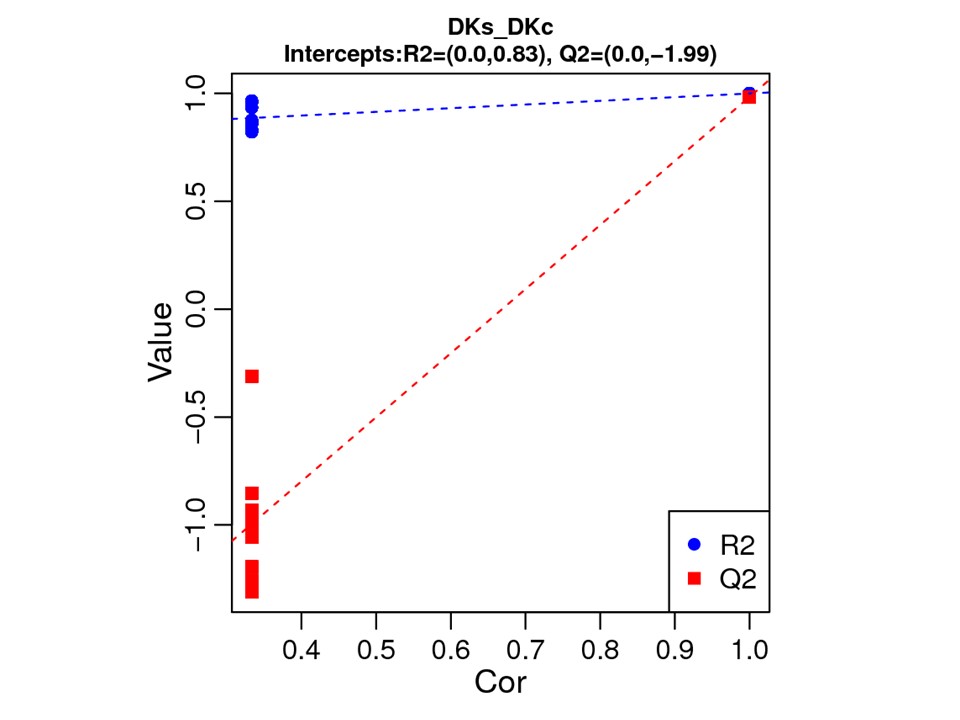

Supplement: Supplementary Figure 3 — The PLS-DA ranking test. [file Image_3.JPEG]

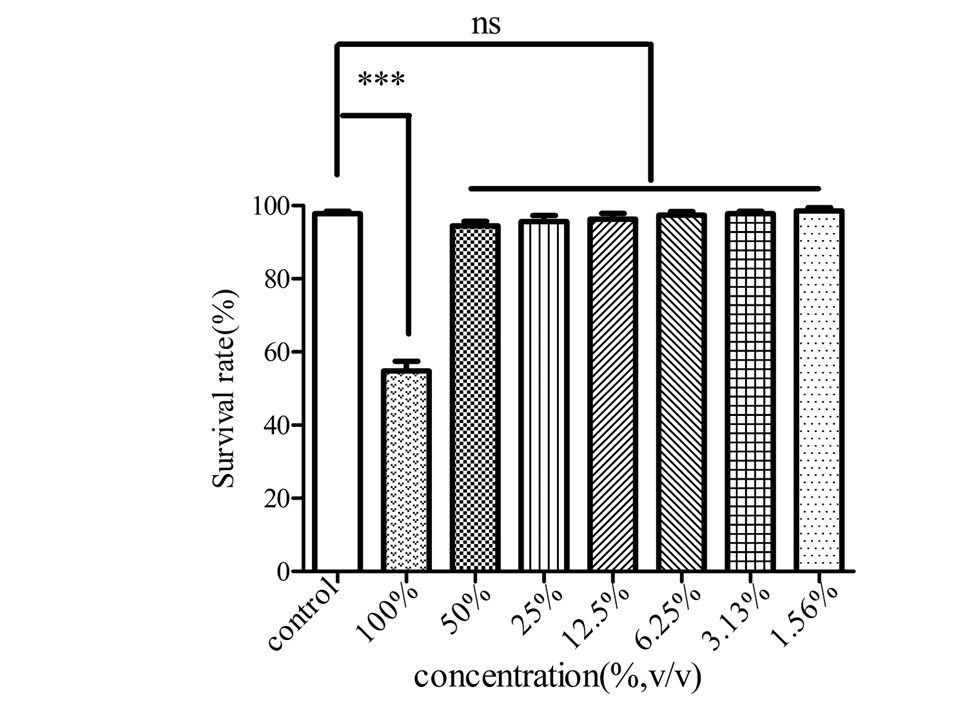

Supplement: Supplementary Figure 4 — Effects of FB on the survival rate of wild-type C. elegans. Differences compared to the control group were considered significant at p < 0.0001 (****) and ns (not significance differences). [file Image_4.JPEG]

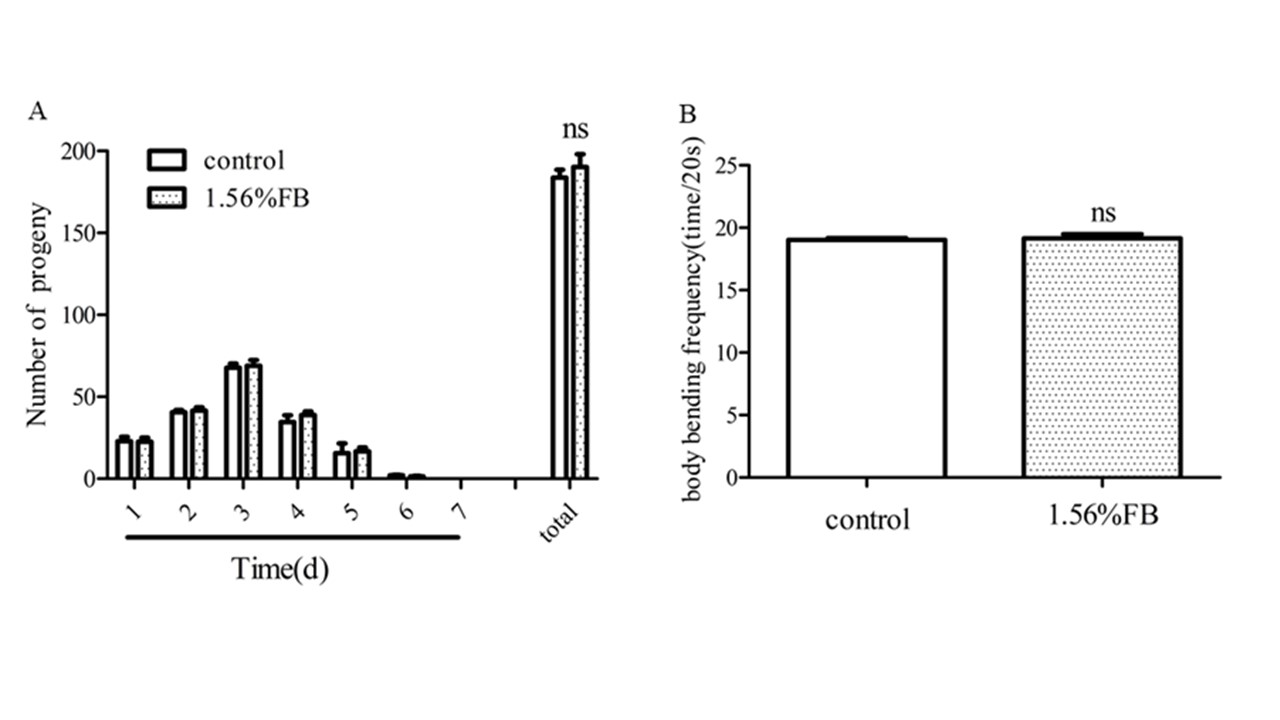

Supplement: Supplementary Figure 5 — The effects of FB on the number of progeny (A) and body bending (B) of C. elegans. No significant (ns) difference compared with the control group. [file Image_5.JPEG]

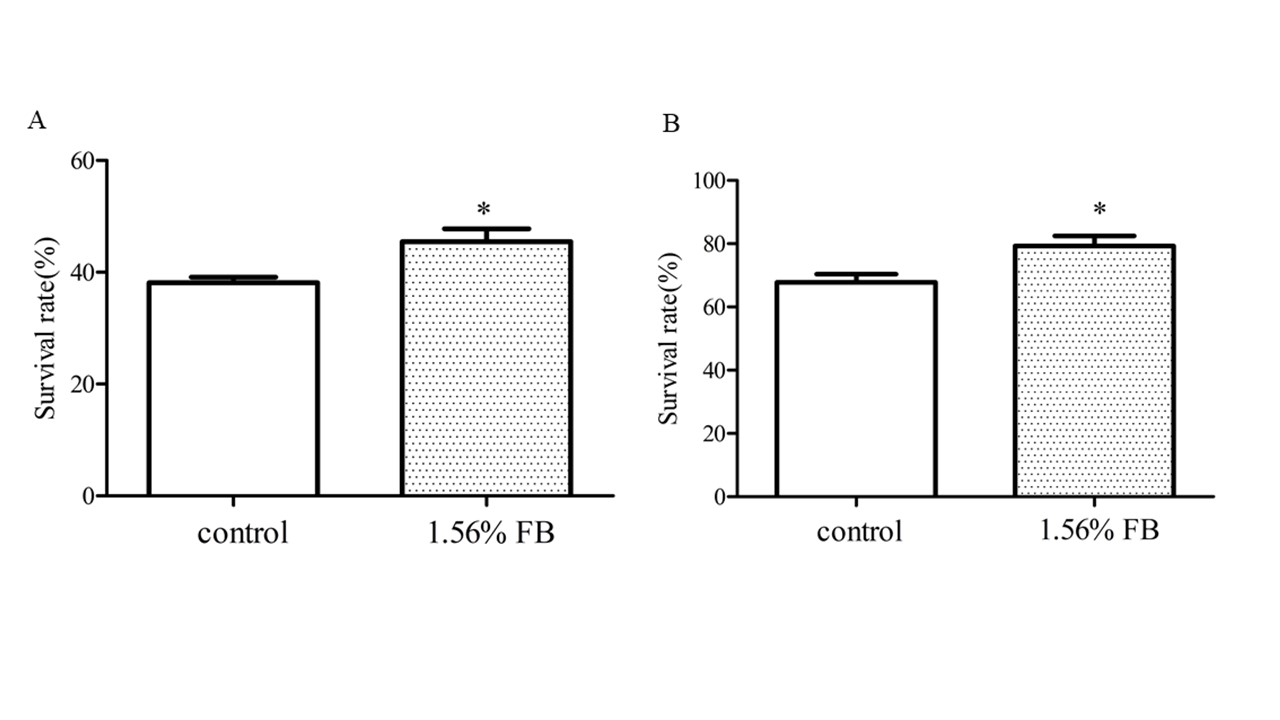

Supplement: Supplementary Figure 6 — Effect of Oxidative stress (A) and heat stress (B) on survival rate of C. elegans. Differences compared to the control group were considered significant at p < 0.05(*). [file Image_6.JPEG]
